# Supplementary material for: HIVprotI: an integrated web based platform for prediction and design of HIV proteins inhibitors
Source: J Cheminform. 2018 Mar 9;10:12. doi: 10.1186/s13321-018-0266-y (PMC5845081; doi:10.1186/s13321-018-0266-y)
Supplement: Supplementary file 2 — Additional file 2. Source code of HIVProtI web server. [file 13321_2018_266_MOESM2_ESM.zip › HIVprotI_Source-code/hivprotI/weka-3-6-6/doc/weka/gui/experiment/ExperimenterDefaults.html]

ExperimenterDefaults


---


|  |  |  |
| --- | --- | --- |
| **Overview** \| **Package** \| **Class** \| **Tree** \| **Deprecated** \| **Index** \| **Help** \| **Weka's home** | |  |
| **PREV CLASS**   **NEXT CLASS** | **FRAMES**    **NO FRAMES**     **All Classes** |  |
| SUMMARY: NESTED \| FIELD \| CONSTR \| METHOD | DETAIL: FIELD \| CONSTR \| METHOD |  |


---


## weka.gui.experiment Class ExperimenterDefaults

```
java.lang.Object
  weka.gui.experiment.ExperimenterDefaults
```

**All Implemented Interfaces:**: java.io.Serializable

---

``` public class ExperimenterDefaults extends java.lang.Object implements java.io.Serializable ```

This class offers get methods for the default Experimenter settings in
the props file `weka/gui/experiment/Experimenter.props`.

**Version:**
:   $Revision: 7059 $

**Author:**
:   FracPete (fracpete at waikato dot ac dot nz)

**See Also:**: `PROPERTY_FILE`, Serialized Form

---

| **Field Summary** | |
| --- | --- |
| `static java.lang.String` | `PROPERTY_FILE`             The name of the properties file |


**Constructor Summary**

`ExperimenterDefaults()`


| **Method Summary** | |
| --- | --- |
| `static java.lang.String` | `get(java.lang.String property, java.lang.String defaultValue)`             returns the value for the specified property, if non-existent then the default value. |
| `static java.lang.String` | `getColumn()`             the comma-separated list of attribute names that identify a column |
| `static java.lang.String` | `getComparisonField()`             returns the name of the field used for comparison |
| `static boolean` | `getDatasetsFirst()`             whether datasets or algorithms are iterated first |
| `static java.lang.String` | `getDestination()`             returns the default destination |
| `static java.lang.String` | `getExperimentType()`             returns the default experiment type |
| `static java.lang.String` | `getExtension()`             returns the default experiment extension |
| `static int` | `getFolds()`             returns the number of folds used for cross-validation |
| `static java.io.File` | `getInitialDatasetsDirectory()`             returns the initial directory for the datasets (if empty, it returns the user's home directory) |
| `static int` | `getMeanPrecision()`             returns the default precision for the mean |
| `static java.lang.String` | `getOutputFormat()`             returns the classname of the ResultMatrix class, responsible for the output format |
| `static java.util.Properties` | `getProperties()`             returns the associated properties file |
| `static boolean` | `getRemoveFilterClassnames()`             whether the filter classnames in the dataset names are removed by default |
| `static int` | `getRepetitions()`             returns the number of repetitions to use |
| `static java.lang.String` | `getRow()`             the comma-separated list of attribute names that identify a row |
| `static boolean` | `getShowAverage()`             returns whether the Average is shown by default |
| `static boolean` | `getShowStdDevs()`             returns whether StdDevs are shown by default |
| `static double` | `getSignificance()`             returns the default significance |
| `static java.lang.String` | `getSorting()`             returns the default sorting (empty string means none) |
| `static int` | `getStdDevPrecision()`             returns the default precision for the stddevs |
| `static java.lang.String` | `getTester()`             returns the display name of the preferred Tester algorithm |
| `static double` | `getTrainPercentage()`             returns the training percentage in case of splits |
| `static boolean` | `getUseClassification()`             whether classification or regression is used |
| `static boolean` | `getUseRelativePaths()`             whether relative paths are used by default |
| `static void` | `main(java.lang.String[] args)`             only for testing - prints the content of the props file |

**Methods inherited from class java.lang.Object**

`equals, getClass, hashCode, notify, notifyAll, toString, wait, wait, wait`

**Field Detail**

### PROPERTY\_FILE

```
public static final java.lang.String PROPERTY_FILE
```

:   The name of the properties file

    **See Also:**: Constant Field Values


**Constructor Detail**

### ExperimenterDefaults

```
public ExperimenterDefaults()
```


**Method Detail**

### get

```
public static java.lang.String get(java.lang.String property,
                                   java.lang.String defaultValue)
```

:   returns the value for the specified property, if non-existent then the
    default value.

    :   **Parameters:**: `property` - the property to retrieve the value for: `defaultValue` - the default value for the property **Returns:**: the value of the specified property

---


### getProperties

```
public static final java.util.Properties getProperties()
```

:   returns the associated properties file

    :   **Returns:**: the props file

---


### getExtension

```
public static final java.lang.String getExtension()
```

:   returns the default experiment extension

    :   **Returns:**: the extension (incl. dot)

---


### getDestination

```
public static final java.lang.String getDestination()
```

:   returns the default destination

    :   **Returns:**: the destination

---


### getExperimentType

```
public static final java.lang.String getExperimentType()
```

:   returns the default experiment type

    :   **Returns:**: the type

---


### getUseClassification

```
public static final boolean getUseClassification()
```

:   whether classification or regression is used

    :   **Returns:**: true if classification

---


### getFolds

```
public static final int getFolds()
```

:   returns the number of folds used for cross-validation

    :   **Returns:**: the number of folds

---


### getTrainPercentage

```
public static final double getTrainPercentage()
```

:   returns the training percentage in case of splits

    :   **Returns:**: the percentage (0-100)

---


### getRepetitions

```
public static final int getRepetitions()
```

:   returns the number of repetitions to use

    :   **Returns:**: the repetitions/runs

---


### getDatasetsFirst

```
public static final boolean getDatasetsFirst()
```

:   whether datasets or algorithms are iterated first

    :   **Returns:**: true if datasets are iterated first

---


### getInitialDatasetsDirectory

```
public static final java.io.File getInitialDatasetsDirectory()
```

:   returns the initial directory for the datasets (if empty, it returns
    the user's home directory)

    :   **Returns:**: the directory

---


### getUseRelativePaths

```
public static final boolean getUseRelativePaths()
```

:   whether relative paths are used by default

    :   **Returns:**: true if relative paths are used

---


### getTester

```
public static final java.lang.String getTester()
```

:   returns the display name of the preferred Tester algorithm

    :   **Returns:**: the display name **See Also:**: `Tester`, `PairedCorrectedTTester`

---


### getRow

```
public static final java.lang.String getRow()
```

:   the comma-separated list of attribute names that identify a row

    :   **Returns:**: the attribute list

---


### getColumn

```
public static final java.lang.String getColumn()
```

:   the comma-separated list of attribute names that identify a column

    :   **Returns:**: the attribute list

---


### getComparisonField

```
public static final java.lang.String getComparisonField()
```

:   returns the name of the field used for comparison

    :   **Returns:**: the comparison field

---


### getSignificance

```
public static final double getSignificance()
```

:   returns the default significance

    :   **Returns:**: the significance (0.0-1.0)

---


### getSorting

```
public static final java.lang.String getSorting()
```

:   returns the default sorting (empty string means none)

    :   **Returns:**: the sorting field

---


### getShowStdDevs

```
public static final boolean getShowStdDevs()
```

:   returns whether StdDevs are shown by default

    :   **Returns:**: true if stddevs are shown

---


### getShowAverage

```
public static final boolean getShowAverage()
```

:   returns whether the Average is shown by default

    :   **Returns:**: true if the average is shown

---


### getMeanPrecision

```
public static final int getMeanPrecision()
```

:   returns the default precision for the mean

    :   **Returns:**: the decimals of the mean

---


### getStdDevPrecision

```
public static final int getStdDevPrecision()
```

:   returns the default precision for the stddevs

    :   **Returns:**: the decimals of the stddevs

---


### getOutputFormat

```
public static final java.lang.String getOutputFormat()
```

:   returns the classname of the ResultMatrix class, responsible for the
    output format

    :   **Returns:**: the classname **See Also:**: `ResultMatrix`, `ResultMatrixPlainText`

---


### getRemoveFilterClassnames

```
public static final boolean getRemoveFilterClassnames()
```

:   whether the filter classnames in the dataset names are removed by default

    :   **Returns:**: true if filter names are removed

---


### main

```
public static void main(java.lang.String[] args)
```

:   only for testing - prints the content of the props file

    :   **Parameters:**: `args` - commandline parameters - ignored


---


|  |  |  |
| --- | --- | --- |
| **Overview** \| **Package** \| **Class** \| **Tree** \| **Deprecated** \| **Index** \| **Help** \| **Weka's home** | |  |
| **PREV CLASS**   **NEXT CLASS** | **FRAMES**    **NO FRAMES**     **All Classes** |  |
| SUMMARY: NESTED \| FIELD \| CONSTR \| METHOD | DETAIL: FIELD \| CONSTR \| METHOD |  |


---
